# Double Trouble: Prevalence and Factors Associated with Tuberculosis and Diabetes Comorbidity in Bangladesh

**DOI:** 10.1371/journal.pone.0165396

**Published:** 2016-10-31

**Authors:** Malabika Sarker, Mrittika Barua, Fiona Guerra, Avijit Saha, Afzal Aftab, A. H. M. Mahbub Latif, Shayla Islam, Akramul Islam

**Affiliations:** 1 James P. Grant School of Public Health, BRAC University, 68, Shaheed Tajuddin Ahmed Sharani, icddr,b Building, Level 6, Mohakhali, Dhaka, 1212, Bangladesh; 2 Public Health Ontario, 480 University Avenue, Suite 300, Toronto, ON M5G 1V2, Canada; 3 Institute of Statistical Research and Training (ISRT), University of Dhaka, Dhaka, 1000, Bangladesh; 4 BRAC, 75 Mohakhali, Dhaka, 1212, Bangladesh; Indian Institute of Technology Delhi, INDIA

## Abstract

**Background:**

Diabetes among tuberculosis patients increases the risk of tuberculosis treatment failure, death, and development of multidrug-resistant tuberculosis. Yet, there is no data is available in Bangladesh on the prevalence of diabetes among tuberculosis patients. The objective of the current study was to estimate prevalence and identify factors associated with tuberculosis-diabetes co-morbidity among TB patients enrolled in the Directly Observed Treatment, Short course program.

**Methods:**

A community based cross-sectional quantitative study was conducted among 1910 tuberculosis patients living in six urban and eleven rural areas among whom Oral Glucose Tolerance Test (those who fasted) and Random Blood Sugar test (those who did not fast) were performed. Besides glucose levels, data on socio-demographic information, family history of diabetes and anthropometric measurements (height and weight) were also collected.

**Result:**

Among the 1910 TB patients who participated in screening for diabetes, 245 (12.8%) were found to have diabetes and 296 (15.5%) to have pre-diabetes. Out of those who had diabetes, 34.7% were newly diagnosed through the current study and 65.3% already knew their status. Among those who were found to have prediabetes, 27 (9.1%) had impaired Fasting Blood Glucose (FBG), 230 (77.7%) had Impaired Glucose Tolerance (IGT), and 39 (13.2%) had both Impaired FBG and IGT. Older age, higher BMI, higher education (secondary level and above), being married, participation in less active work, and family history of diabetes are associated with higher prevalence of diabetes.

**Conclusion:**

We observed a higher prevalence of diabetes and pre-diabetes in TB patients than reported previously in Bangladesh among the general population which may challenge TB and diabetes control in Bangladesh. Diabetes diagnosis, treatment and care should be integrated in the National TB Program.

## Introduction

With the increasing pace of globalization, the disease burden in low to middle-income countries (LMICs) is no longer limited to communicable diseases but also non-communicable diseases (NCDs) [1]. More alarming is that the co-existence of both communicable and NCDs amplifies the risk of developing other diseases and can adversely affect treatment outcomes [2]. One unique example of such a combination is tuberculosis-diabetes (TB-diabetes) comorbidity. Globally, 15% of TB patients suffer from diabetes [3]. Patients suffering from TB-diabetes, with poor glycemic control, experience poor TB treatment outcomes such as treatment failure and death among TB patients [4] compared to those with better glycemic control [5]. In addition, TB-diabetes patients are more likely to develop multidrug-resistant TB (MDR-TB) [6], and have higher mortality [7].

The rise of diabetes is one of the greatest threats to LMICs in the South East Asia Region where, approximately 60% of deaths predominantly occur due to chronic NCDs like diabetes [8]. Bangladesh is no exception to this fact. Previous studies in Bangladesh tell us about the increase in prevalence of diabetes from 2005 to 2011 (from 8% to 15% in urban, and from 2% to 8% in rural areas) [9,10]. Such an increase in prevalence of diabetes in a span of six years highlights the increasing probability of higher prevalence of TB among the general population as diabetes makes an individual prone to TB [4].

There is hardly any particular trend in the prevalences of TB reported over the last few years in Bangladesh. The prevalence of TB in Bangladesh, according to the Global Tuberculosis Report for 2013 [11] by the World Health Orgnaization (WHO) is 434 per 100,000. Following this, the next two reports by WHO for 2014 [12] and 2015 [13] reported the prevalences to be 402 per 100,000 and 404 per 100,000 respectively. However, these numbers are not a true representation due to lack of contribution of data as acknowledged in these reports. Nevertheless, such a high prevalence of TB cannot be ignored.

Prevention of TB is already a challenge in Bangladesh, with a growing number of MDR-TB patients. On top of this, a disease like diabetes further increases the probability of TB, particularly MDR-TB, as discussed earlier. This in turn poses a threat to TB control efforts. Although several studies have identified the risk factors of diabetes in Bangladesh [9,10,14,15], until now no study has looked at the prevalence of TB-diabetes comorbidity in Bangladesh and its associated factors. Therefore it is of utmost importance that we measure the TB-diabetes comorbidity prevalence and determine the associated factors to propose cost-effective interventions to tackle this dual morbidity.

The current study aimed to identify prevalence and the associated factors of TB-Diabetes comorbidity among TB patients enrolled in Direct Observed Treatment Shortcourse (DOTS) programme jointly run by National Tuberculosis Programme (NTP) of Bangladesh and Bangladesh Rural Advancement Committee (BRAC) TB Control Programme in Bangladesh.

## Methods

### Study setting

This study was conducted in six purposively selected urban (Dhaka, Sylhet, Barisal, Chittagong, Narayonganj, and Gazipur) and ten rural (Bogra, Noakhali, Dinajpur, Comilla, Narsingdi, Cox’s Bazaar, Kushtia, Sherpur, Manikganj, and Khagrachari) areas of Bangladesh, based on the tuberculosis notification rate (low and high equal for urban and rural) reported by the BRAC TB program. These areas are covered by BRAC, an NGO that works with the National Tuberculosis Programme of Bangladesh, along with 42 other local NGOs in screening and treating TB. More rural areas were selected due to the enrollment of fewer TB patients in the rural DOTS centers compared to the urban areas.

### Study design, population and sample size

This study was a part of a larger community based cross-sectional quantitative study on TB-diabetes comorbidity. The larger study has two objectives. One, to determine the prevalence of diabetes among TB patients and the associated factors. Two, to determine sputum conversion throughout the treatment period to see whether diabetes affects sputum conversion (from Acid Fast Bacilli (AFB) positive to AFB negative). Participants took part in the screening for diabetes and a survey. The sampling frame consisted of patients diagnosed with either pulmonary (PTB) or extra pulmonary TB (EPTB) enrolled in DOTS in the selected areas. The inclusion criteria were: aged 18 years and above and enrolled in DOTS within the last eight weeks, PTB cases confirmed with AFB positive sputum or x-ray or EPTB cases clinically confirmed following the standard diagnostic criteria approved by Bangladesh, and initiation of treatment within the last two months from the day of the screening [16].

A sample of 708 TB subjects was required for each category of rural and urban area, which was calculated using the 25% prevalence of diabetes among TB patients in India, a neighboring country which is socio-culturally similar to Bangladesh [17], 80% power, 5% level of significance, and non-response rate of 20%. In total 1911 patients (779 for urban area and 1132 for rural area) were recruited in the study. All patients were referred to the diagnostic facilities by BRAC health workers, and received a transport allowance and snacks after the test. All tests were provided free of cost.

All patients except one who voluntarily came to the diagnostic centers took part in screening of diabetes. Although all patients were advised to fast before coming for the test, they were still asked if they fasted. Those who fasted underwent Oral Glucose Tolerance Test (OGTT) which included measurement of blood sugar during fasting and two hours after administration of food (for those who knew their diabetes status) or 75 g glucose solution (for those who did not know their diabetes status). Blood drawn during fasting measured fasting glucose, and the one drawn two hours after measured glucose tolerance. For the OGTT, the following categories adopted by World Health Organization (WHO) were used (Table 1) [18].

**Table 1:** Categories for Oral Glucose Tolerance Test adopted by World Health Organization [18].

Stages	Fasting	2 hr post glucose load
Normal	<6.1 mmol/l	<7.8 mmol/l
Impaired Fasting Glucose	6.1–<7.0 mmol/l	<7.8 mmol/l
Impaired Glucose Tolerance	<7.0 mmol/l	7.8–<11.1 mmol/l
Diabetes Mellitus	> = 7.0 mmol/l	> = 11.1 mmol/l

We adopted the following definitions in our study:

- Non-diabetes: When blood glucose for fasting and 2 hour post glucose load meets criteria for “normal”, then it is non-diabetes.
- Pre-diabetes: We assigned pre-diabetes when either of these came true:
  ○ A person has Impaired Fasting Glucose (IFG) and a normal glucose tolerance
  ○ A person has normal fasting glucose but an Impaired Glucose Tolerance (IGT)
  ○ A person has both IFG and IGT.
- Diabetes: We assigned diabetes when either of these came true:
  ○ Glucose level for fasting is normal but for glucose tolerance is > = 11.1mmol/l
  ○ Glucose level for fasting is impaired but for gluocose tolerance is > = 11.1 mmol/l
  ○ Glucose level for fasting is > = 7.0 mmol/l and for glucose tolerance is > = 11.1 mmol/l
  ○ A person self reported having diabetes.

We also decided to determine diabetic status of those who delivered blood to measure Fasting Blood Glucose (FBG) only. In this case we decided to use criteria from the “fasting” column of Table 1. The ones who did not fast were tested for Random Blood Sugar (RBS) only, where criteria for 2 hour post glucose load (from Table 1) were used as reference. We asked them what they had before coming for the test and at what time they had it. We maintained the two hour waiting period for them as OGTT.

In case of BMI, we used a cut-off point for overweight in our study that is different from the existing WHO cut-off point for overweight. This is because, according to a WHO Expert Consultation [19], Asian people with high risk of diabetes and cardiovascular diseases have BMIs lower than the existing WHO cut-off point for overweight. It has been observed that the risk varies from 22 kg/m^2^ to 31 kg/m^2^. Hence there is no single cut-off point. Therefore we used BMI cut off point for overweight as > = 23 kg/m^2^ with reference to a review article on overview of obesity by Ashrafuzzaman [20].

### Sampling methods

**Fig 1:**
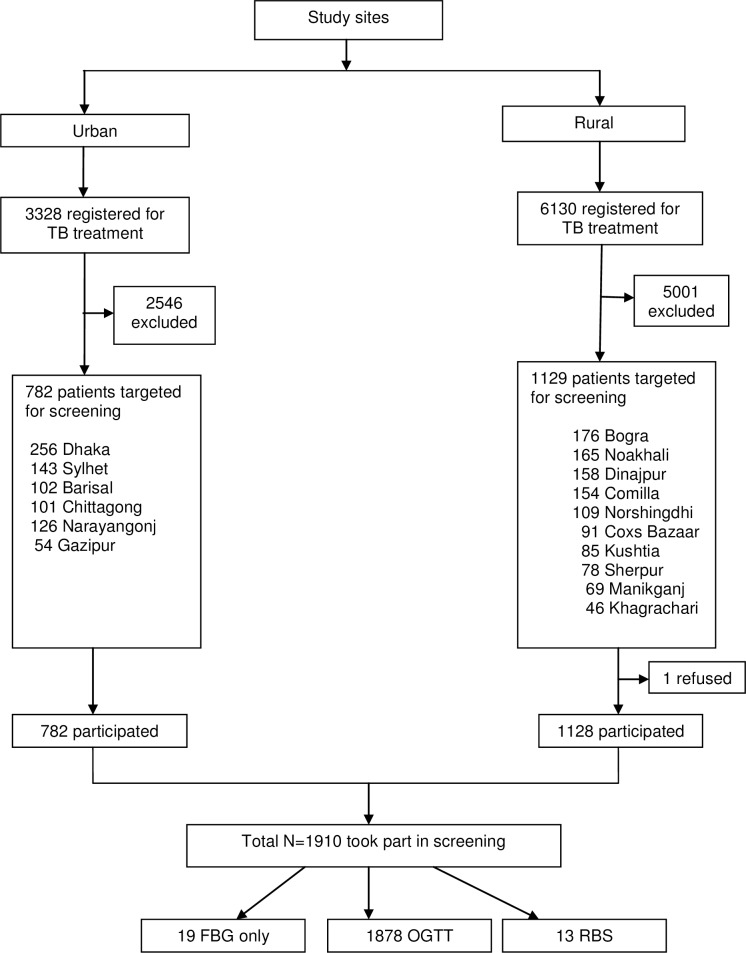
Schematic representation of number of participants screened from the study sites. TB = Tuberculosis; OGTT = Oral Glucose Tolerance Test; RBS = Random Blood Sugar; FBG = Fasting Blood Glucose

We recruited the patients with the support from Program Organizers working in the BRAC TB program. A list of all TB patients was collected from the BRAC TB Program registers in the study sites. Out of 9458 TB patients registered for DOTS under BRAC in the selected study sites, 7547 TB patients did not meet the inclusion criteria and thus were excluded. The number of TB patients eligible for screening was 1911 out of which one refused to participate due to fear of blood to be drawn. Therefore, 1910 TB patients were screened (Fig 1).

The patients were contacted the day before the diabetes screening by BRAC TB Program organizers over telephone and/or through household visits by *Shasthya Shebikas* (front line community health workers). The patients were explained the purpose and procedure of the screening method. They were infomed that they would undergo screening to determine their diabetic status. They were instructed to fast before coming for the test and that this test would be voluntary and free of cost. With the help of BRAC TB Program organizers, the researchers contacted the diagnostic centers affliated with BRAC where screening took place. The personnel at the diagnostic centers were informed about OGTT and the number of patients they could expect. Only the patients who came to the diagnostic centers were screened for diabetes.

### Data Collection

The TB patients came early in the morning with their treatment cards to the designated diagnostic centers. Data collection took place in two phases: collection of blood and face-to-face interview using a pre-tested questionnaire. After arrival of the patients, researchers explained the purpose of the study and the procedure of OGTT to each participant, and took their verbal consent and medical history of diabetes. All patients except one agreed to participate in the screening and the survey. The one who refused to undergo screening due to fear of blood to be drawn left the center.

In phase one, blood was drawn twice among those who fasted: first time immediately after arrival, and second time two hours after administration of food (among those who reported having diabetes), or 75g oral glucose solution (among those who did not know their diabetes status). The first blood drawn was to measure FBG and the second blood sample was drawn to measure Glucose Tolerance (GT). Blood was drawn once to measure RBS among those who did not fast before coming for the test.

In phase two, data on family history of diabetes, demographic and socioeconomic information, and anthropometric measurements (height and weight) were collected by the researchers. Details of smear results of sputum tests were taken from treatment cards. The test results of the diabetes screening were collected later in the evening on the same day or next day of the blood glucose test, photocopied, and kept in a secure location with the researchers. The original test results were put in a sealed envelope and handed over to BRAC TB Program organizers or *Shasthya Shebikas* to be distributed to the TB patients and refer them for counselling and treatment wherever appropriate.

### Ethical approval

Ethical approval was obtained from the Bangladesh Medical Research Council, National Research Ethics Comitteee before starting the study. Verbal consent was taken from all patients as many were illiterate and signing or providing fingerprint on a paper is a sensitive issue. Oral and written information about the nature and purpose of the study were provided to all study participants before verbal consent was taken. The participants were also informed that their participation was voluntary and could withdraw from the study anytime. The interviewer read the Bangla consent form to the study participant for their approval and cross checked the "yes" option in the questionnaire. The ERC committee was aware about the cultural context and approved this consent procedure. The TB patients who were diagnosed with prediabetes or newly diagnosed with diabetes were referred to hospitals and clinics for further investigation and management of diabetes.

### Statistical Analysis

Statistical Package for Social Science (SPSS) was used for data entry and cleaning and STATA (version 12.0) was used for all statistical analysis. The outcome variable was the presence of diabetes or pre-diabetes among TB patients. Descriptive univariate analysis was conducted to determine the proportions of different categorical variables, and means and standard deviations of continuous variables. Bivariate analyses such as chi-squared tests were conducted to identify factors associated with diabetes status so that only the significant factors (p< = 0.10) could be added in the final regression model. Since the outcome variable (diabetes status) has three categories (no diabetes, pre-diabetes, and diabetes), multinomial logistic regression was used to identify factors associated with TB with diabetes and pre-diabetes comorbidity.

## Results

Even though all TB patients were instructed to fast before coming for the screening, not all of them fasted. Out of 1910 TB patients, 1897 patients fasted. Among those who fasted, 19 gave blood sample for FBG only and 1878 gave blood samples for both FBG and GT. This means that the 13 patients who did not fast were tested for RBS. The ones who had their blood drawn for FBG only left the diagnostic centres after first blood was drawn as they did not want to wait for 2 hours.

### Socio-demographic profile

The mean age (including standard deviation) of the participants was 39.9 (± 15.5) years. Among the participants, 38.7% were females, 53.1% had a family income of 5000–10,000 BDT, 40.7% never attended school, 79.5% were married, 44.4% were unemployed, and 59.2% were from rural areas (Table 2).

**Table 2:** Comparison of study characteristics among TB patients screened for diabetes from July 2013 to March 2014.

		Diabetic Status		
Characteristics	Total N = 1910	Non-diabetes	Pre-diabetes	Diabetes
		(n = 1369)	(n = 296)	(n = 245)
**Age (Years)**				
< 30 years	616 (32.2)	513 (37.5)	81 (27.4)	22 (8.9)
30–44 years	534 (28.0)	384 (28.1)	69 (23.3)	81 (33.1)
45–59 years	445 (23.3)	278 (20.3)	82 (27.7)	85 (34.7)
> = 60 years	315 (16.5)	194 (14.2)	64 (21.6)	57 (23.3)
Mean (± SD)	39.9 ± 15.5	38.0 ± 15.2	42.6 ± 16.4	47.3 ± 13.6
**Sex**				
Male	1170 (61.3)	816 (59.6)	193 (65.2)	161 (65.7)
Female	740 (38.7)	553 (40.4)	103 (34.8)	84 (34.3)
**Educational Status**				
Never attended school	778 (40.7)	568 (41.5)	131 (44.3)	79 (32.3)
Primary education	478 (25.1)	349 (25.5)	65 (21.9)	64 (26.1)
Secondary education	379 (19.8)	268 (19.6)	60 (20.3)	51 (20.8)
SSC and above	275 (14.4)	184 (13.4)	40 (13.5)	51 (20.8)
**Marital Status**				
Never Married	279 (14.6)	223 (16.3)	48 (16.2)	8 (3.2)
Married	1519 (79.5)	1069 (78.1)	234 (79.1)	216 (88.2)
Widow/Widower/Divorced/Separated	112 (5.9)	77 (5.6)	14 (4.7)	21 (8.6)
**Employment status**				
Unemployed	848 (44.4)	612 (44.7)	137 (46.3)	99 (40.4)
Employed	940 (49.2)	691 (50.5)	132 (44.6)	117 (47.8)
Retired	122 (6.4)	66 (4.8)	27 (9.1)	29 (11.8)
**Monthly Family Income (BDT)**				
<5000	329 (17.2)	240 (17.5)	55 (18.6)	34 (13.9)
5000 to 10,000	1088 (57.0)	796 (58.2)	162 (54.7)	130 (53.1)
>10,000	493 (25.8)	333 (24.3)	79 (26.7)	81 (33.0)
**Site type**				
Rural	1131 (59.2)	816 (59.6)	173 (58.4)	142 (58.0)
Urban	779 (40.8)	553 (40.4)	123 (41.6)	103 (42.0)
**TB classification**				
Pulmonary TB	1570 (82.2)	1122 (82.0)	241 (81.4)	207 (84.5)
Extra-pulmonary TB	331 (17.3)	241 (17.6)	53 (17.9)	37 (15.1)
Unknown	9 (0.5)	6 (0.4)	2 (0.7)	1 (0.4)
**Type of TB cases**				
New cases	1876 (98.2)	1348 (98.5)	292 (98.7)	236 (96.3)
Previously treated	34 (1.8)	21 (1.5)	4 (1.3)	9 (3.7)
**Type of occupation**				
Highly active	551 (28.9)	417 (30.5)	84 (28.4)	50 (20.4)
Moderately active	868 (45.4)	646 (47.2)	121 (40.9)	101 (41.2)
Less active	491 (25.7)	306 (22.3)	91 (30.7)	94 (38.4)
**BMI**				
Normal (18.50–22.99)	1004 (52.6)	744 (54.4)	166 (56.1)	94 (38.4)
Underweight (<18.50)	672 (35.2)	481 (35.1	90 (30.4)	101 (41.2)
Overweight/obese (> = 23.00)	234 (12.2)	144 (10.5)	40 (13.5)	50 (20.4)
**Family History of Diabetes**				
Absent	1770 (92.7)	1287 (94.0)	275 (92.9)	208 (84.9)
Present	140 (7.3)	82 (6.0)	21 (7.1)	37 (15.1)

BMI = Body Mass Index; TB = Tuberculosis; SSC = Secondary School Certificate

### Clinical profile with regards to TB

Among the participants, 1570 (82.2%) had pulmonary TB and 331 (21.8%) had EPTB. The type of TB (PTB or EPTB) among the remaining nine (0.5%) participants was unknown. These nine participants did not have their treatment cards with them. They were recently enrolled in treatment regimen and had their information recorded in the program registers at the BRAC TB Program offices but not on treatment cards at the time of data collection. The DOTS program organizers confirmed the eligibility of patients who did not present their treatment cards. Out of the ones who had pulmonary TB, 75.7% were confirmed through sputum and 24.3% through x-ray (Table 2).

### Prevalence of diabetes and pre-diabetes among study participants

Among the 1910 TB patients who participated in screening for DM, 245 (12.8%) were found to have diabetes and 296 (15.5%) to have pre-diabetes (Table 2). Out of those who had diabetes, 34.7% were newly diagnosed through the current study and 65.3% already knew their diabetes status. Among those who were found to have prediabetes, 27 (9.1%) had impaired FBG, 230 (77.7%) had IGT, and 39 (13.2%) had both Impaired FBG and IGT.

Among the 160 TB patients who knew their diabetes status, 12 (7.5%) were not engaged in any type of intervention such as exercise, diet change, or medicine (tablet, insulin) to control their diabetes.

Among the participants who were found to have diabetes, the majority belonged to the age group of 45–59 years. More individuals were identified with pre-diabetes (44.3%) and diabetes (32.3%) among those who never attended school compared to the ones who had at least some level of education. The prevalence of diabetes was higher in rural areas than urban areas among the TB patients with diabetes (58.0% VS 42.0%). The prevalences of prediabetes and diabetes were higher among individuals with monthly family income between 5000 to 10000 BDT compared to individuals with family income of less than 5000 BDT (Table 2).

In the current study, the majority of the participants had BMI between 18.50 and 22.99 kg/m^2^. Fifty (20.4%) TB patients with diabetes were overweight (> = 23.0 kg/m^2^) compared to 144 (10.5%) TB patients with no diabetes. Most of the participants were also involved in moderately active occupation and had no family history of diabetes. However, among the 245 individuals with diabetes, only 50 (20.4%) had a highly active occupation compared to 101 (41.2%) and 94 (48.4%) individuals who were involved in moderately or less active occupations respectively. With respect to family history of diabetes, 37 (15.1%) participants with family history of diabetes had diabetes, compared to 21 (7.1%) and 82 (6.0%) pariticipants who were found to have pre-diabetes or no diabetes respectively (Table 2).

### Factors associated with TB-diabetes comorbidity

Age, sex, BMI, education, employment status, marital status, type of site (rural or urban), type of occupation, and family history of diabetes were found to be associated with diabetes status through chi-squared analysis (p = <0.10) and included in the final logistic regression model. All these variables except sex were found to be significantly associated with diabetes status (Table 3).

**Table 3:** Multinomial logistic regression analysis for factors associated with pre-diabetes and diabetes among study subjects.

	Number of TB patients screened for diabetes	Pre-diabetes	Diabetes
	N = 1910	n = 296	n = 245
	n (%)	OR (95% CI)	OR (95% CI)
**Age**			
< 30 years	616 (32.2)	-	-
30–44 years	534 (28.0)	1.39 (0.93–2.08)	4.73 (2.77–8.09)***
45–59 years	445 (23.3)	2.56 (1.65–3.98)***	8.72 (4.89–15.52)***
> = 60 years	315 (16.5)	2.54 (1.54–4.20)***	8.67 (4.55–16.52)***
**Sex**			
Male	1170 (61.3)	-	-
Female	740 (38.7)	0.97 (0.66–1.43)	0.86 (0.54–1.39)
**Educational Status**			
Never attended school	778 (40.7)	-	-
Primary education	478 (25.1)	0.95 (0.67–1.34)	1.71 (1.16–2.52)**
Secondary education	379 (19.8)	1.19 (0.81–1.75)	2.23 (1.44–3.44)***
SSC and above	275 (14.4)	1.01 (0.64–1.61)	2.63 (1.63–4.27)***
**Marital Status**			
Never Married	279 (14.6)	-	-
Married	1519 (79.5)	0.68 (0.44–1.05)	2.66 (1.18–5.99)*
Widow/Widower/Divorced/	112 (5.9)	0.41 (0.19–0.91)*	3.39 (1.21–9.46)*
Separated			
**Employment status**			
Unemployed	848 (44.4)	-	-
Employed	940 (49.2)	0.68 (0.49–0.94)*	0.78 (0.52–1.17)
Retired	122 (6.4)	1.09 (0.63–1.89)	1.23 (0.68–2.20)
**Monthly Family Income (BDT)**			
<5000	329 (17.2)	-	-
5000 to 10,000	1088 (57.0)	0.91 (0.64–1.30)	1.15 (0.75–1.78)
>10,000	493 (25.8)	1.06 (0.70–1.59)	1.46 (0.90–2.36)
**Site type**			
Rural	1131 (59.2)	-	-
Urban	779 (40.8)	1.19 (0.90–1.57)	1.18 (0.86–1.62)
**Type of occupation**			
Highly active	551 (28.9)	-	-
Moderately active	868 (45.4)	0.94 (0.63–1.40)	1.29 (0.78–2.12)
Less active	491 (25.7)	1.47 (1.03–2.09)*	1.83 (1.20–2.78)**
**BMI**			
Normal (18·50–22·99)	1004 (52.6)	-	-
Underweight (<18·50)	672 (35.2)	0.85 (0.63–1.13)	1.45 (1.05–2.02)*
Overweight/obese (> = 23·00)	234 (12.2)	1.30 (0.86–1.95)	2.09 (1.36–3.21)**
**Family History of Diabetes**			
None of the parents	1770 (92.7)	-	-
At least one parent	140 (7.3)	1.33 (0.79–2.23)	3.06 (1.89–4.96)***

*P values between 0.05–0.01

**P values between 0.01–0.001

***P values less than 0.001

BMI = Body Mass Index; TB = Tuberculosis; SSC = Secondary School Certificate

In multinomial logistic regression, several factors were associated with being diagnosed with diabetes and pre-diabetes. Both diabetes and pre-diabetes were significantly associated with older age. It was revealed that TB patients aged more than 30 years are at significantly higher risk of having diabetes than those aged less than 30 years. Higher chances of diabetes have been found to be associated with an education level of SSC and above with an odds ratio of 2.63 (95% C.I 1.63–4.27). The regression model further determined that married TB patients are 2.66 (95% C.I 1.18–5.99) times more likely to have diabetes than those who are never married (Table 3).

## Discussion

To the best of our knowledge, this is the first study in Bangladesh that reports the prevalence of diabetes and pre-diabetes among TB patients. The current study aimed to determine the prevalence of diabetes and pre-diabetes among TB patients and the factors associated with TB-diabetes comorbidity. The prevalences of diabetes and pre-diabetes were found to be 12.8% and 15.5% respectively. The factors significantly associated with TB-diabetes comorbity were age (more than 30 years), education level of SSC and above, being married, less active occupation, higher BMI, and presence of family history of diabetes.

The prevalence of diabetes among TB patients reported in this study is lower than that reported in India in 2012 [17] (prevalence of diabetes being 25.3%), which shares socio-economic similarities with Bangladesh. However, we observed a higher prevalence of diabetes in the TB population than in the general population of Bangladesh as reported from a nationwide survey [10] (prevalence: 9.7%) and a systemic review [21] (pooled prevalence: 6.7%). The higher prevalence of diabetes found in our study could be due to our study design where we actively screened people from the community. The high prevalence of diabetes can also be attributed to TB status as diabetes can weaken the immune system and make patients prone to TB [22].

Moreover, the current study reported a higher number of individuals with diabetes in the rural areas compared to Akter et al (10). One reason could be the poor screening of diabetes in rural areas.

We also observed a lower prevalence of pre-diabetes (15.5%) in our study compared to the one by Akter et al (15.5% VS 23.0%) [10] [. This suggests that there may be an increased risk of diabetes in Bangladesh in the future. Overall, the alarming increases of diabetes and pre-diabetes indicate a threat to TB control and demands a need of increasing awareness regarding lifestyle changes.

The study participants with diabetes and pre-diabetes were significantly older than participants with no diabetes or pre-diabetes. The association of increasing age with diabetes evident from the study is consistent with past studies conducted in India [17,23,24] and China [25]. Increasing age has been established to be associated with both TB and diabetes through previous studies [26,27].

In the current study, TB patients with lower BMI have been observed to have a lower risk of diabetes than those with higher BMI. From the literature, we do know that lower BMI and diabetes make an individual more prone to TB [28]. This means with increasing BMI, although the risk of diabetes increases, the risk of TB decreases [29]. But the increasing prevalence of diabetes, as evident through our study, suggests more occurrence of TB, hence raising the co-morbidity of TB and diabetes [30]. Therefore, the importance of undertaking interventions to reduce TB-diabetes comorbidity cannot be neglected.

We also observed that family history of diabetes has a strong significant association with occurrence of diabetes. This means those who have family history of diabetes are at more risk of having diabetes than those who do not. Our study reports a considerably higher number of individuals with TB-diabetes comorbidity with family history of diabetes which is not surprising. This is consistent with studies conducted in Bangladesh [31] and other parts of the world as well [16,20,21]

Among the study participants diagnosed with diabetes, around 65% of them were previously diagnosed, indicating 35% were newly diagnosed through our study. It has already been established that diabetes acts as a risk factor for TB as it can worsen the immune response needed to prevent occurrence of TB. This could be a reason for the higher prevalence of diabetes in the current study. The other reason could be that we screened TB patients who were already on treatment. The literature suggests that TB induces hyperglycemia and mimics diabetes in non-diabetic patients [32]. In addition, post initiation of anti-tubercular treatment, TB drugs such as rifampicin and isoniazid may also induce hyperglycemia by enhancing the metabolism of hypoglycemic agents in anti-diabetes drugs, and impairing insulin secretion in non-diabetics [32,33]. It may even worsen glucose control in diabetes patients, which may require changes in the dose of insulin [34]. Thus, over-diagnosis of diabetes in TB patients might occur if screening for glucose is not conducted prior to initiation or after finishing TB treatment. All the respondents in our study were screened for diabetes after initiation of TB treatment that could lead to over-diagnosis. However, this provides an opportunity for the patients to receive an intervention, earlier on, for better control and treatment outcomes, if/when needed. More research should be conducted to find the ideal time for diabetes screening. Glucose levels should be measured repeatedly before, during and after completion of TB treatment to determine true diabetic status.

The strengths of the study comprise the large number of TB patients screened for diabetes reducing selection bias in the process, determining prevalence of both diabetes and pre-diabetes among TB patients in Bangladesh, using OGTT method to screen diabetes, and covering urban and rural areas. However, limitations should also be mentioned. We could not perform OGTT among all the patients. We performed RBS among those who did not fast and only FBG among those who did not complete OGTT. Tests such as RBS and FBG have lower sensitivity than OGTT [23,35]. There is a possibility of underestimating the prevalence of diabetes and pre-diabetes among those who took part to measure RBS and FBG only. Even though OGTT was a strength for the current study, it also acted as a limitation. For an OGTT, it is required to fast for 8 hours before giving blood for FBS, and further requires the individual to wait for 2 hours before the blood can be drawn again to measure GT. Such strict requirements become a burden for a sick person such as a TB patient. In addition, the timing of screening posed a problem as well. Most of the participants in our study needed to go to work early morning. While some could not complete the OGTT procedure, some had to take leave and/or report late to work, causing further mental and physical stress. In addition, our study findings can only be generalized for patients diagnosed with TB but not to other vulnberable populations. Last but not the least, recruiting TB patients on treatment raises a possibility of overestimating the prevalence of diabetes, as previously mentioned.

As diabetes is a risk factor for TB, an ideally effective TB control program could expand and incorporate diabetes care among individuals with active TB; however funding is a challenge. Therefore, co-management of both the diseases could be limited to active TB patients only [36]. The World Health Organization along with the International Union Against Tuberculosis and Lung Diseases developed a collaborative framework for care and control of TB and diabetes, which highly recommends bidirectional screening for both [37].

The importance of screening for diabetes in TB patients cannot be ignored. In low-resource countries, there is a lack of screening of diabetes and inadequate diabetes control. But diabetes is rising gradually due to socioeconomic and lifestyle changes [38]. The relative contribution of diabetes to TB epidemic will be 12·5% in 2030, an increase of 25·5% compared to 2010. In countries with a high TB burden, in particular, the risk for TB is three times more in diabetic patients [38]. In LMICs, TB patients are usually not screened for diabetes as a part of the management of TB control, partly due to cost and complexity [39]. For this reason, different screening approaches have been proposed, including diabetes risk assessment surveys, and use of suitable, rapid, inexpensive screening tools appropriate for TB patients in LMICs. Countries like Bangladesh depend on OGTT as the gold standard to detect diabetes, but this method requires a clinical setting with a long waiting time and multiple blood samples. Other screening methods such as point-of-care glycated haemoglobin and glycated albumin assays, and sudomotor function-based screening devices are currently being explored [39].

The high prevalence of pre-diabetes in the current study underscores the importance for preventive measures for diabetes. Factors revealed in this study may increase the risk of diabetes along with other factors like use of tobacco, unhealthy diets, and lack of physical activities [10,14]. Tuomilehto et al. in 2001 followed a cohort of glucose-impaired subjects for three years and observed decreased incidence of diabetes due to changes in lifestyle [40].

Nevertheless, awareness regarding TB- diabetes comorbidity should be raised among the DOTS implementers. Tuberculosis control programs in Bangladesh should incorporate routine screening of diabetes in TB patients and refer them to diabetes care facilities. Such an undertaking would require training for capacity building, as well as improvement in infrastructure. This means a greater collaboration must take place between the National Tuberculosis Program Bangladesh and the Diabetic Association of Bangladesh (DAB) to ensure guidelines are revised to respond to the dual burden.

In conclusion, the current study reported a high prevalence of diabetes and pre-diabetes among TB patients which may challenge TB and diabetes control in Bangladesh. Therefore future research should focus on reducing TB and diabetes comorbidity.
